# Myocardial perfusion and oxygenation are impaired during stress in severe aortic stenosis and correlate with impaired energetics and subclinical left ventricular dysfunction

**DOI:** 10.1186/1532-429X-16-29

**Published:** 2014-04-29

**Authors:** Masliza Mahmod, Jane M Francis, Nikhil Pal, Andrew Lewis, Sairia Dass, Ravi De Silva, Mario Petrou, Rana Sayeed, Stephen Westaby, Matthew D Robson, Houman Ashrafian, Stefan Neubauer, Theodoros D Karamitsos

**Affiliations:** 1 Division of Cardiovascular Medicine, Radcliffe Department of Medicine, John Radcliffe Hospital, University of Oxford Centre for Clinical Magnetic Resonance Research (OCMR), Oxford OX3 9DU, UK; 2 Department of Cardiothoracic Surgery, Oxford University Hospitals, Oxford OX3 9DU, UK

**Keywords:** Hypertrophy, Myocardial ischaemia, Microvascular dysfunction, Oxygenation, Energetics, Strain

## Abstract

**Background:**

Left ventricular (LV) hypertrophy in aortic stenosis (AS) is characterized by reduced myocardial perfusion reserve due to coronary microvascular dysfunction. However, whether this hypoperfusion leads to tissue deoxygenation is unknown. We aimed to assess myocardial oxygenation in severe AS without obstructive coronary artery disease, and to investigate its association with myocardial energetics and function.

**Methods:**

Twenty-eight patients with isolated severe AS and 15 controls underwent cardiovascular magnetic resonance (CMR) for assessment of perfusion (myocardial perfusion reserve index-MPRI) and oxygenation (blood-oxygen level dependent-BOLD signal intensity-SI change) during adenosine stress. LV circumferential strain and phosphocreatine/adenosine triphosphate (PCr/ATP) ratios were assessed using tagging CMR and ^31^P MR spectroscopy, respectively.

**Results:**

AS patients had reduced MPRI (1.1 ± 0.3 vs. controls 1.7 ± 0.3, p < 0.001) and BOLD SI change during stress (5.1 ± 8.9% vs. controls 18.2 ± 10.1%, p = 0.001), as well as reduced PCr/ATP (1.45 ± 0.21 vs. 2.00 ± 0.25, p < 0.001) and LV strain (−16.4 ± 2.7% vs. controls −21.3 ± 1.9%, p < 0.001). Both perfusion reserve and oxygenation showed positive correlations with energetics and LV strain. Furthermore, impaired energetics correlated with reduced strain. Eight months post aortic valve replacement (AVR) (n = 14), perfusion (MPRI 1.6 ± 0.5), oxygenation (BOLD SI change 15.6 ± 7.0%), energetics (PCr/ATP 1.86 ± 0.48) and circumferential strain (−19.4 ± 2.5%) improved significantly.

**Conclusions:**

Severe AS is characterized by impaired perfusion reserve and oxygenation which are related to the degree of derangement in energetics and associated LV dysfunction. These changes are reversible on relief of pressure overload and hypertrophy regression. Strategies aimed at improving oxygen demand–supply balance to preserve myocardial energetics and LV function are promising future therapies.

## Background

Aortic valve stenosis (AS) is characterized by pressure overload left ventricular hypertrophy (LVH) and impaired myocardial perfusion reserve [1]. This has been shown by a variety of imaging modalities such as positron emission tomography (PET) [2] and cardiovascular magnetic resonance (CMR) [3]. In the absence of significant coronary stenoses, this finding is indicative of microvascular dysfunction, but it remains unclear whether the hypoperfusion seen in severe AS leads to myocardial tissue deoxygenation and, thus, ischemia during stress. Such knowledge is important as myocardial tissue hypoxia may play a significant pathophysiological role in the natural history of AS contributing to the development of LV dysfunction.

CMR provides the unprecedented capability to assess myocardial perfusion *and* oxygenation in the same setting during vasodilator stress [4]. Assessment of myocardial perfusion reserve during vasodilator stress following the first pass of a gadolinium-based contrast agent is an established CMR technique [5,6]. Oxygenation-sensitive CMR can non-invasively assess myocardial tissue oxygenation without the need for exogenous contrast by measuring blood-oxygen level-dependent (BOLD) signal intensity (SI) differences, which reflect deoxygenated hemoglobin concentration during adenosine stress [7-10]. Oxygenation measurements using BOLD CMR have been shown to be proportional to changes in coronary sinus oxygen saturation, [7] and are affected in patients with microvascular dysfunction as well [9]. Thus, compared to perfusion, myocardial oxygenation may be a superior parameter reflecting more directly the imbalance between oxygen demand and supply that characterizes ischemia.

Previous studies have shown that the hypertrophied heart such as in AS shows impaired myocardial energetics demonstrated by a reduced ratio of phosphocreatine to adenosine triphosphate (PCr/ATP) [11,12]. However the relationship between oxygenation and myocardial energy metabolism in AS has not been investigated yet. ^31^P magnetic resonance (MR) spectroscopy is a noninvasive technique that can be used to study myocardial energy metabolism in vivo.

Therefore, the purpose of the present study was to comprehensively assess coronary microvascular status in patients with severe AS and no obstructive CAD by assessing myocardial perfusion *and* oxygenation during adenosine stress. We hypothesized that tissue oxygenation and perfusion during stress are impaired in severe AS and correlate with myocardial energy metabolism and LV contractile function. We also hypothesized that these abnormalities are restored after aortic valve replacement (AVR). If our hypotheses are proven true, it would suggest a central role of myocardial ischemia in patients with severe AS despite the absence of epicardial coronary stenoses.

## Methods

### Study population

Thirty-four patients with isolated severe AS were prospectively recruited from the Oxford University Hospital National Health Service Trust. Six patients were excluded (1 had HCM, 1 had poor LV function, 1 had severe claustrophobia and 3 were unable to undergo adenosine stress). Of the 28 patients, 3 patients were asymptomatic and 25 had at least one of typical symptoms consistent with severe aortic valve stenosis (angina, breathlessness or syncope). All patients had undergone invasive coronary angiography, showing unobstructed epicardial coronary arteries. Selection criteria included an aortic valve area of ≤1.0 cm^2^, peak gradient of 64 mmHg without other significant valvular pathology based on clinical reports of echo performed as part of routine clinical evaluation, systolic blood pressure (BP) <160 mmHg and diastolic BP <90 mmHg. Patients who had LVEF <50%, contraindications to MR imaging, glomerular filtration rate <60 ml/min, underlying cardiomyopathy, previous myocardial infarction, coronary revascularization or previous cardiac surgery were excluded. Of the 25 AS patients (symptomatic) who underwent AVR, 14 were rescanned 8 months after AVR. Eleven patients did not have a follow-up scan (2 died perioperatively, 1 had pacemaker implantation, 5 were lost to follow-up and 3 did not consent for a repeat CMR). Fifteen healthy volunteers (defined by no history of heart disease, diabetes, hypertension or high cholesterol, not taking any medications, normal physical examination and electrocardiogram) served as controls.

### Study protocol

All subjects underwent baseline CMR scanning on a 3 Tesla MR system (TIM Trio; Siemens Healthcare, Erlangen, Germany) within 4–6 weeks of the routine clinical echocardiogram. Fasting venous blood was drawn for blood glucose and lipid profile. All subjects gave their informed consent to participate in the study which was approved by the National Research Ethics Service Committee South Central - Berkshire.

### Cardiac magnetic resonance protocol

Study participants were instructed to avoid caffeine-containing food and drinks for at least 24 hours prior to CMR. Cine imaging was performed using standard methods [13]. Strain imaging was performed using myocardial tagging sequence as previously described [14]. A short-axis image at the mid ventricular level was acquired. ^31^P MR spectroscopy was performed to obtain the PCr/ATP ratio from a voxel placed in the midventricular septum, with the subjects lying prone with their heart over the centre of the ^31^P heart/liver coil in the iso-centre of the magnet as previously described [15].

BOLD and stress perfusion CMR were performed as previously described [16,17]. Briefly, adenosine (140 μg/kg/min) was infused for at least 3 minutes, followed by acquisition of a single mid-ventricular slice (identical to the one acquired at rest) of BOLD image. Then stress perfusion imaging was performed immediately after stress BOLD imaging, using a bolus a gadolinium-based contrast (Gadodiamide, Omniscan; GEHealthcare) at a dose of 0.03 mmol/kg followed by a saline flush. After discontinuing adenosine, rest perfusion images were acquired 20 minutes after the stress study to allow sufficient time for contrast washout. Furthermore, after rest perfusion imaging, an additional bolus of 0.1 mmol/kg gadodiamide was administered (total dose: 0.16 mmol/kg) for late gadolinium enhancement (LGE) imaging. The LGE images were acquired as previously described [18].

### Cardiac magnetic resonance data analysis

Analysis of cardiac volumes, function and mass was performed as previously described [13]. All analyses were performed in a blinded fashion. Aortic valve area was measured from direct planimetry during aortic valve cine CMR. Information on the peak aortic valve gradient was obtained from the clinical echocardiogram performed during work-up for AVR. LV circumferential strain was analysed by using CIM-Tag software (Auckland, New Zealand) [19]. ^31^P MRS post processing analysis was performed as previously described [15].

BOLD analysis was performed as described previously [16]. In brief, myocardial SI was measured after manually tracing the endocardial and epicardial contours. The mid-ventricular short-axis BOLD image was divided into 6 segments. Mean signal intensities were calculated for resting and stress conditions by averaging signal measurements from images during rest and adenosine stress, respectively.

For perfusion analysis, briefly, 3 contours (epicardial, endocardial and LV blood pool) were drawn on a single image and propagated throughout the perfusion series. The myocardium was divided into segments based on the American Heart Association (AHA) segmentation model. SI versus time curves were generated and normalized to the LV blood pool upslope. Myocardial perfusion reserve index (MPRI) was defined as the ratio of stress to rest relative upslope [20].

LGE was only deemed to be present when the area of contrast enhancement could be seen in both phase-swapped images and in a cross-cut long-axis image. LGE quantification was performed using cmr42 software (version 4.0, Circle Cardiovascular Imaging). The full-width-half-maximum (FWHM) technique was used to quantify fibrosis as previously described [21]. Fibrosis volume was expressed as the LGE mass (g) and also as a percentage of total myocardial mass.

### Statistical analysis

All data are expressed as mean ± standard deviation and checked for normality using Kolmogorov–Smirnov test. Categorical data are presented as numbers and percentages. Comparisons between the 2 groups were performed by Student *t*-test. The chi-square test or Fisher’s exact test was used to compare discrete data as appropriate. Bivariate correlations were performed using Pearson’s or Spearman’s method as appropriate. Comparisons between pre- and post-AVR measurements in AS patients were performed with two-tailed paired *t*-test. Comparisons between AS patients and controls at baseline and post-AVR were performed using independent Student *t*-test or Mann–Whitney U test as appropriate. A *P*-value <0.05 was considered significant. All statistical analyses were performed with IBM SPSS Statistics, version 20.

## Results

### Baseline study characteristics

Demographic, clinical, echocardiographic and biochemical data are shown in Table 1. All AS patients had both AVA of ≤1.0 cm^2^ and peak gradient of ≥64 mmHg based on echocardiogram performed as part of routine clinical evaluation. The AS patients were slightly older than the healthy volunteers but numerically the difference was small (69 vs. 63 years). There were no significant differences in gender, body mass index, BP and pulse rate between AS patients and normal controls. Blood glucose and lipid parameters were also similar in both groups.

**Table 1:** Baseline characteristics of patients and normal controls

	**Severe AS (n = 28)**	**Normal controls (n = 15)**	**P value**
Age (years)	69 ± 10	63 ± 4	0.01
Male, n (%)	21 (75)	8 (53)	0.16
Body mass index (kg/m^2^)	28 ± 5	27 ± 4	0.41
Systolic blood pressure (mmHg)	134 ± 17	131 ± 11	0.52
Diastolic blood pressure (mmHg)	75 ± 9	76 ± 9	0.52
Heart rate (bpm)	66 ± 10	62 ± 9	0.18
Peak aortic valve gradient (mmHg)*	82 ± 14	−	
Symptoms, n (%)			
Asymptomatic	3 (11)	−	
Dyspnoea	18 (64)	−	
Angina	8 (29)	−	
Syncope	1 (<1)	−	
Past medical history, n (%)			
Hypertension	11 (39%)	−	
Dyslipidaemia	7 (25%)	−	
Diabetes	5 (18%)	−	
Medications, n (%)			
Aspirin	11 (39)	−	
Metformin	5 (18)	−	
ACE-I/ARB-II	8 (29)	−	
Beta-blockers	5 (18)	−	
Statin	13 (46)	−	
Biochemical (mmol/L)			
Blood glucose	5.5 ± 1.7	5.1 ± 0.9	0.36
Total cholesterol	4.6 ± 1.0	5.5 ± 0.6	0.013
Triglycerides	1.2 ± 0.8	1.0 ± 0.3	0.58
High-density lipoprotein	1.4 ± 0.4	1.5 ± 0.4	0.83

Values are mean ± SD or percentages. ACE indicates angiotensin-converting enzyme-inhibitors; *ARB*, Angiotensin-receptor antagonist-II. *Based on echocardiographic measurement.

### Assessment of left ventricular mass, function, myocardial fibrosis and energetics

The CMR results are summarized in Table 2. As expected, AS patients had significant LVH, reduced circumferential strain, high normal LVEF and reduced PCr/ATP ratio when compared to the controls. LGE imaging demonstrated that myocardial fibrosis was prevalent in AS (79%) and none was found in the normal controls. There was patchy mid-wall enhancement pattern affecting predominantly the basal inferior and inferolateral wall. None of the AS patients had any evidence of previous myocardial infarction on LGE imaging. By using the FWHM method for LGE quantification, the burden of LV fibrosis was high in AS, 33.2 g (17.1-61.8) or 19.7 ± 11.0% of total LV mass.

**Table 2:** CMR results in AS patients vs. normal controls

	**Aortic stenosis (n = 28)**	**Normal (n = 15)**	**P value**
BOLD signal intensity change (%)	5.1 ± 8.9	18.2 ± 10.1	0.001
Myocardial perfusion reserve index	1.1 ± 0.3	1.7 ± 0.3	<0.001
Circumferential strain (%)	−16.4 ± 2.7	−21.3 ± 1.9	<0.001
PCr/ATP	1.45 ± 0.21	2.00 ± 0.25	<0.001
LV ejection fraction (%)	74 ± 6	69 ± 4	0.01
LV end-diastolic volume (ml)	142 ± 45	137 ± 33	0.73
LV end-systolic volume (ml)	37 ± 20	42 ± 12	0.43
LV mass index (g/m^2^)	95 ± 30	56 ± 13	<0.001
Aortic valve area (cm^2^)	0.84 ± 0.10	4.08 ± 0.73	<0.001
LGE present, n (%)	22 (79)	-	-
LGE volume when positive			
Mass (g)	33.2 (17.1-61.8)	-	-
Percentage myocardium (%)	19.7 ± 11.0	-	-

Values are mean ± SD, percentages or median (interquartile range). *BOLD*, Blood oxygen level dependent; *LV*, Left ventricular; *LGE*, Late gadolinium enhancement.

### Assessment of myocardial perfusion reserve and oxygenation under adenosine stress

**Figure 1:**
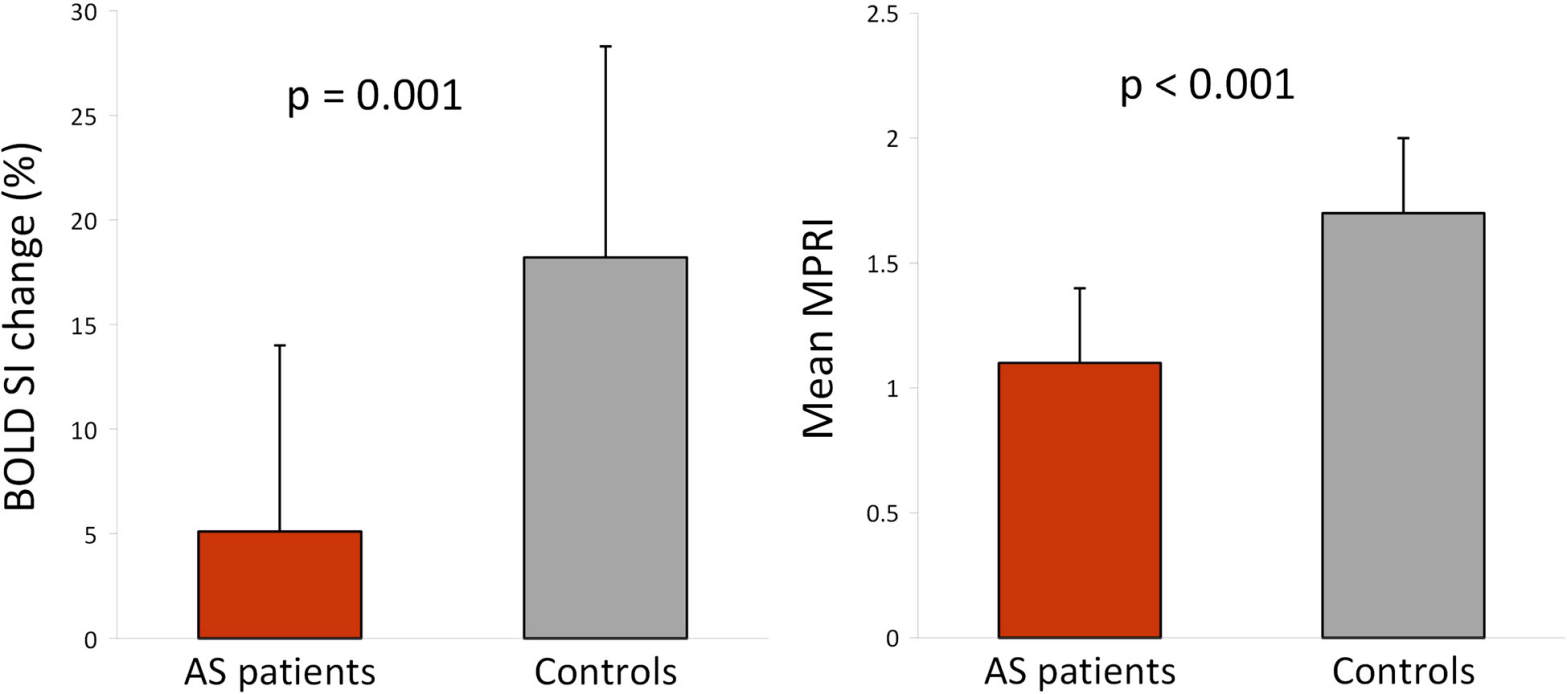
**BOLD SI change and MPRI in AS patients when compared to normal controls.** Error bars represent standard deviation.

During adenosine stress, there were equivalent rises in rate pressure product (RPP) in both AS patients and controls; baseline RPP 8.6 ± 1.6×10^3^ (beats/min).mmHg in AS vs 8.0 ± 1.5×10^3^ (beats/min).mmHg in controls, p = 0.27 and stress RPP 11.2 ± 2.5×10^3^ (beats/min).mmHg in AS vs 11.7 ± 2.6×10^3^ (beats/min).mmHg in controls, p = 0.55. Confirming previous CMR and PET studies [2,3], myocardial perfusion reserve was significantly reduced in AS patients compared to the healthy controls. Importantly, we now report that oxygenation is also substantially reduced in AS patients compared to normal controls, as shown in Table 2 and Figure 1.

### Relationships amongst oxygenation, perfusion, strain and energetics

**Figure 2:**
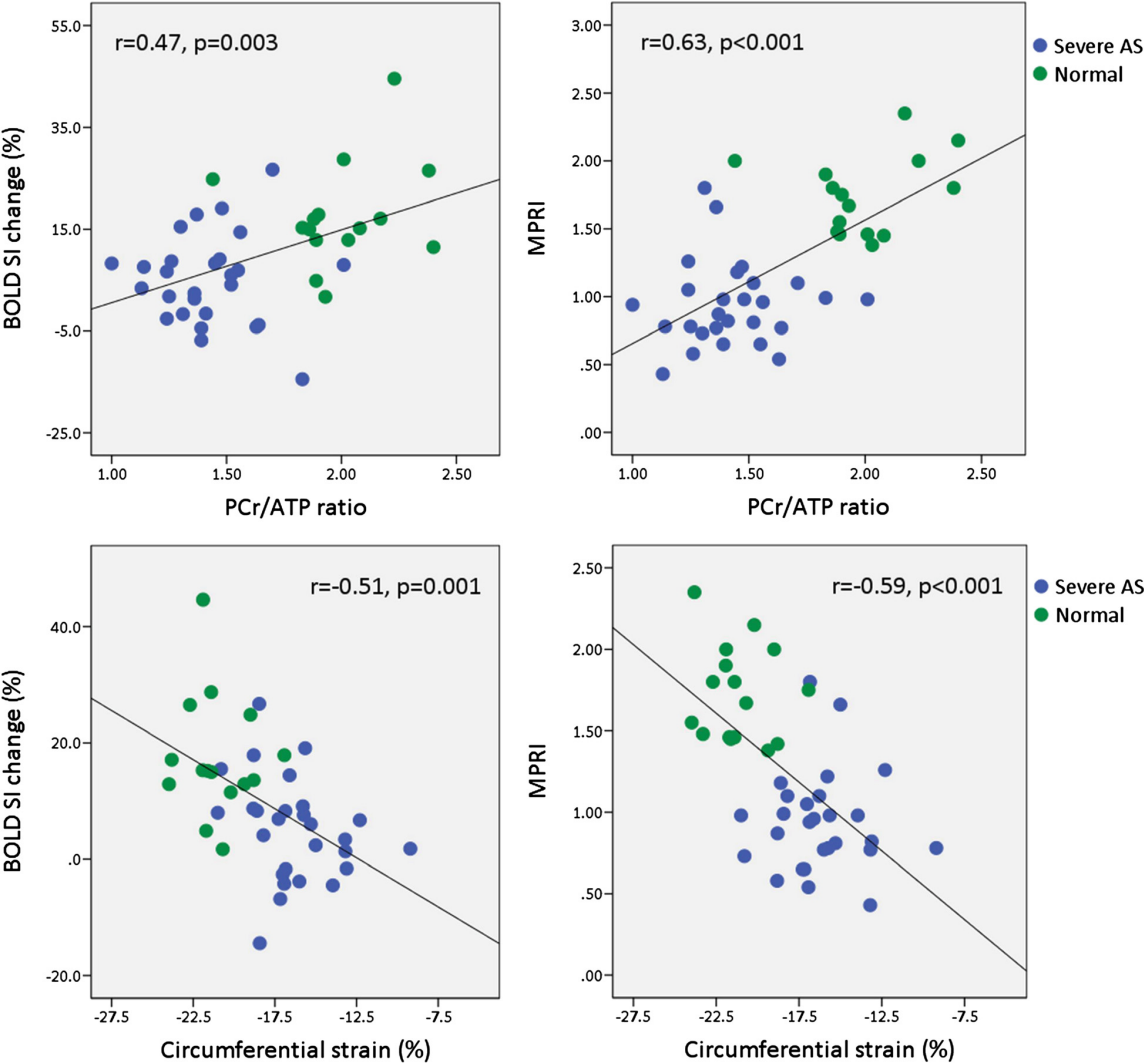
Correlations between BOLD SI change and MPRI with PCr/ATP ratio (top panel) and circumferential strain (bottom panel).

In line with previous studies, we found that MPRI had an inverse correlation with LVMI (r = −0.61, p < 0.001), presence of LGE (Rs = −0.61, p < 0.001) but not with LGE volume or LGE%, and a positive correlation with AVA (r = 0.45, p = 0.02) [2,3]. Furthermore, we also found that impaired MPRI was associated with blunting of myocardial oxygenation during vasodilator stress (r = 0.52, p = 0.001), impaired strain (r = −0.59, p < 0.001) and reduced PCr/ATP (r = 0.63, p < 0.001) (Figure 2). Similar to perfusion, BOLD SI change had an inverse correlation with LVMI (r = −0.39, p = 0.02), circumferential strain (r = −0.51, p = 0.001) and a positive correlation with PCr/ATP (r = 0.47, p = 0.003), but no significant correlation with presence of LGE, LGE volume or LGE%. We also found that PCr/ATP ratio inversely correlated with strain (r = −0.75, p < 0.001). LGE volume (g) also increased with increasing LVMI (r = 0.72, p < 0.001).

### CMR and spectroscopy post AVR

**Figure 3:**
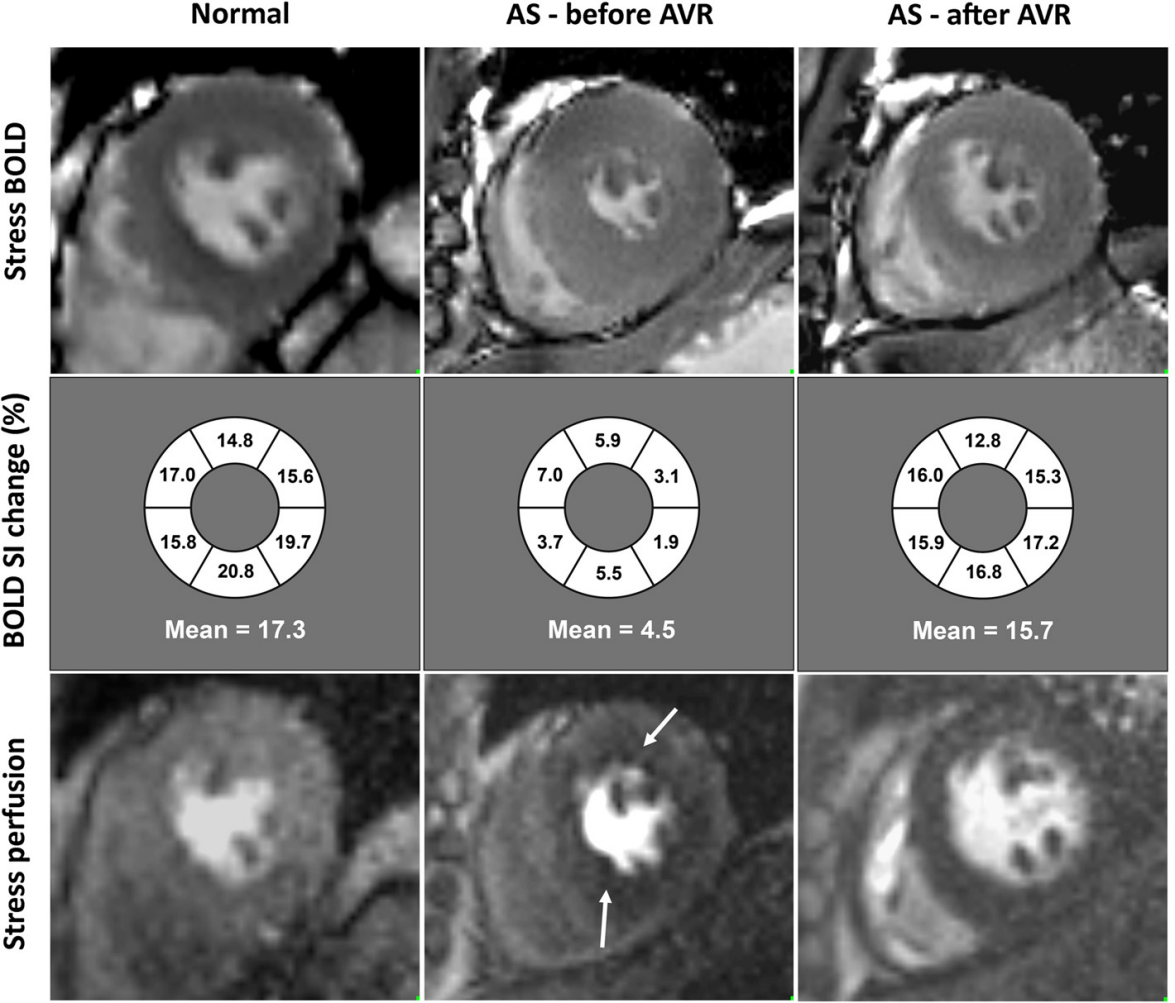
**Examples of BOLD imaging.** From top to bottom panels, short-axis stress BOLD, schematic diagram of segmental BOLD SI change, and stress perfusion. From left to right, normal volunteer, a patient with severe aortic stenosis (AS) before and after aortic valve replacement (AVR). Note the circumferential perfusion defect in the AS patient pre AVR (white arrows), which is no longer present after AVR.

Results are summarized in Table 3 with an example shown in Figure 3. In accordance with previous studies, we confirmed significant improvements in perfusion [22], LV strain [23] and energetics [24] after AVR. We have now shown that there was also significant improvement in oxygenation post AVR with no significant difference between the post-AVR levels and normal controls. Although LGE volume (g) reduced following AVR, LGE% of myocardium was similar to the baseline level. Thus, abnormalities in perfusion, LV strain, energetics and oxygenation were all reversible on relief of LV overload and hypertrophy regression.

**Table 3:** Cardiac MRI and spectroscopy before and after AVR

	**All AS patients (n = 28)**	**Follow-up AS patients (n = 14)**		**Controls**
		**Pre AVR**	**Post AVR**	
BOLD signal intensity change (%)	5.1 ± 8.9	5.1 ± 8.4	15.6 ± 7.0*	18.2 ± 10.1
Myocardial perfusion reserve index	1.1 ± 0.3	1.0 ± 0.4	1.6 ± 0.5*	1.7 ± 0.3
Circumferential strain (%)	−16.4 ± 2.7	−16.3 ± 2.3	−19.4 ± 2.5*	−21.3 ± 1.9
PCr/ATP	1.45 ± 0.21	1.42 ± 0.17	1.86 ± 0.48*	2.00 ± 0.25
Left ventricular mass index (g/m^2^)	95 ± 30	98 ± 31	69 ± 17†	56 ± 13
Left ventricular ejection fraction (%)	74 ± 7	76 ± 5	74 ± 5^‡^	69 ± 4
LGE volume				
Mass (g)	33.2 (17.1-61.8)	29.5 (16.7-54.2)	17.7 (15.1-33.9)**	-
Percentage myocardium (%)	19.7 ± 11.0	18.1 ± 10.7	17.2 ± 10.8***	-

Values are mean ± SD or median (interquartile range). ATP indicates adenosine triphosphate; *BOLD*, Blood oxygen level-dependent; *LGE*, Late gadolinium enhancement; *PCr*, Phosphocreatine.

*p < 0.05 vs pre AVR and >0.05 vs controls; †p < 0.05 vs pre AVR and controls; ‡p > 0.05 vs pre AVR and <0.05 vs controls; **p < 0.05 vs pre AVR; ***p > 0.05 vs pre AVR.

We further explored the data by comparing the baseline characteristics, severity of AS and CMR findings of the AS patients with (n = 14) and without follow-up (n = 11). Patients who did not have follow-up CMR were older than those who had follow-up CMR after AVR (73 vs. 65 years), in which the 3 patients who had perioperative complications (2 deaths and 1 pacemaker implantation) were among the oldest in that group. The rest of the baseline characteristics and severity of AS were similar in both groups (results not shown). The CMR indices of patients without follow-up CMR scan, in particular BOLD SI change (5.0 ± 11.2%), MPRI (0.9 ± 0.2), circumferential strain (−16.0 ± 3.3%), PCr/ATP ratio (1.43 ± 0.25), LVMI (90 ± 27 g/m^2^), LVEF (74 ± 7%), LV mass (33.0 [18.5-49.5]) and %LV mass (17.9 ± 6.8%) were also similar to the CMR indices of the patients with follow-up scan, all p > 0.05.

## Discussion

There are two main findings of this study. First, we demonstrated that significant impairment in myocardial perfusion *and* oxygenation response to vasodilator stress in severe AS patients and normal epicardial coronaries, which correlate with the degree of impairment in myocardial energetics and contractility. Second, following AVR, alterations in myocardial perfusion and oxygenation are reversible along with improvements in energetics and circumferential strain.

### Myocardial perfusion and oxygenation in AS

Microvascular dysfunction is a well-established phenomenon in pressure-overload LVH [25]. This can be due to decreased capillary density per unit myocardium due to inadequate growth of new vessels and extravascular compression as a result of increased LV wall stress [25,26]. We demonstrate that hypoperfusion seen is severe enough to be translated into a significantly blunted myocardial oxygenation response to stress. Not surprisingly, our data show that the degree of impairment in perfusion reserve correlates with the extent of hypertrophy, aortic valve stenosis and myocardial fibrosis.

In the absence of obstructive CAD, the hypertrophied ventricle is vulnerable to ischemia especially under stress [26]. This is thought to be the explanation why patients with AS have symptoms of effort angina despite normal epicardial coronary arteries [27]. Whether de-oxygenation occurs as a consequence of impaired perfusion reserve during stress in AS has never been demonstrated previously. Myocardial hypoperfusion does not always reflect hypoxia as oxygen demand may vary in different pathophysiologic states. For example, in hypertension, myocardial oxygen utilization per unit LV weight is reduced in the presence of LVH [28]. Cardiac hypertrophy is an adaptive mechanism in response to pressure overload, to normalize increased wall stress according to the Law of Laplace [29]. Our group recently demonstrated that dissociation between myocardial perfusion and oxygenation can exist [9,16,30]. Impaired stress perfusion can be compatible with normal oxygenation when myocardial oxygen demand is down-regulated such as in hibernation [9,16], and stress oxygenation can be reduced in spite of normal perfusion, when the oxygen demand is increased such as in HCM gene carriers [30]. Our current study now shows that, in the context of AS, perfusion and oxygenation during stress are closely associated, and both are impaired. This is in line with observations in patients with LVH due to long-standing hypertension [31]. We also showed that both perfusion and oxygenation impairments in AS showed significant associations with the degree of LVH. Conversely, there was no significant relationship between oxygenation and fibrosis, which is expected because scar tissue has negligible oxygen requirements.

### Relationships amongst myocardial perfusion/oxygenation, energetics and contractility

Our results confirm the previous finding of reduced PCr/ATP ratio in patients with severe AS [11,12]. We now demonstrated that impaired perfusion reserve and oxygenation correlate with impaired energetics and reduced circumferential strain. Impaired energetics is likely to be due to microvascular dysfunction [24] or reduced total creatine [32], or both, as known to occur in the hypertrophied heart. ATP is the direct energy fuel for myocardial contraction whilst phosphocreatine is essential for the transfer of high-energy phosphates from mitochondria to myofibrils. Whether the relationship between energetics and function in the hypertrophied and failing heart is causal or coincidental is a highly complex [33] and controversial [34], and our study cannot resolve this.

### Changes following aortic valve replacement

We showed that improvement in oxygenation 8 months after AVR occurred in parallel with improvements in stress perfusion [22], energetics [24] and LV strain [19]. This improvement in microcirculatory function after AVR is most likely the result of a combination of both, relief of mechanical obstruction with reduced LV wall stress, and LVH regression [24,35]. Although there was significant LGE volume reduction after AVR, LGE as a percentage of myocardium did not change. It is likely that LGE reduction was related to LV mass regression as shown by positive correlation between LGE volume and LVMI in our study. As gadolinium is confined to the extracellular space, reduction in LGE may also indicate a reduction of other elements of extracellular matrix and not due to collagen reduction per se. Similarly, other studies have shown no significant fibrosis regression post AVR using diffuse equilibrium [36] and qualitative assessments [37].

### Clinical implications

Our findings suggest that substantial microvascular dysfunction leading to myocardial tissue ischaemia during vasodilator stress is present in severe AS without epicardial CAD. A reduced capacity to augment myocardial oxygenation during stress may contribute to poor clinical outcome, as prognosis rapidly deteriorates after development of symptoms such as angina and the onset of LV dysfunction. Oxygenation-sensitive CMR together with MR-spectroscopy provide a comprehensive insight into the pathophysiology of ischaemia and metabolic derangements in AS patients. Importantly, our findings suggest a potential new avenue for therapy targeted at improving microcirculation and reducing oxygen demand, e.g. nitrates [38], calcium antagonists and beta-blockers, or metabolic agents to improve efficiency of energy utilization e.g. perhexiline [39].

### Study limitations

We included a small number of AS patients with diabetes, hypertension and dyslipidaemia, which may contribute to myocardial perfusion abnormalities. However, only patients with well-controlled diabetes and BP were included and the few patients who had dyslipidaemia were well treated by statin. It would be challenging to recruit patients with severe AS without any of these conditions which are very common in the elderly population. However, the reversibility of our findings after AVR strongly suggests that our findings were not due to concomitant diseases. Although the present BOLD technique only acquired a single mid-ventricular slice, this should not affect the result as left ventricular hypertrophy in AS is general. Our study demonstrates proof of concept, and confirmation of our findings in larger scale studies is warranted.

## Conclusions

In severe AS without epicardial CAD, there is blunted oxygenation response to adenosine stress suggestive of microvascular impairment, which correlates with impaired energetics and subclinical LV dysfunction. Myocardial perfusion, oxygenation, energetics and contractility are restored following AVR. Oxygenation-sensitive CMR provides pathophysiologic insight, may become a helpful diagnostic tool, and suggests novel strategies for therapy in AS aimed at improving the oxygen demand/supply balance.

## Abbreviations

AS: Aortic stenosis; AVR: Aortic valve replacement; BOLD: Blood-oxygen level dependent; CMR: Cardiovascular magnetic resonance; FWHM: Full width half maximum; LGE: Late gadolinium enhancement; LVEF: Left ventricular ejection fraction; LVH: Left ventricular hypertrophy; LVMI: Left ventricular mass index; MPRI: Myocardial perfusion reserve index; PCr/ATP ratio: Phosphocreatine to adenosine triphosphate ratio; SI: Signal intensity.

## Competing interests

The authors declare that they have no competing interests.

## Authors’ contributions

MM: contributed to the conception, design and coordination of the study, substantially acquired and analyzed the data, performed statistical analysis and drafted the manuscript; JMF and NP contributed to data acquisition and critical revision of the manuscript; AL contributed to data analysis and critical revision of the manuscript; SD, RDS, MP, RS, SW, MDR, HA have critically revised the manuscript; SN and TDK participated in the conception and design of the study and critically revised the manuscript. All authors read and approved the final manuscript.
